# Pericarditis Following Recovery From COVID-19 Infection in a 15-Year-Old Boy: A Postinflammatory Immune-Mediated Presentation or a New-Onset Autoimmune Disease

**DOI:** 10.7759/cureus.19255

**Published:** 2021-11-04

**Authors:** Nikolas Lamprinos, Fani Ladomenou, Sofia Stefanaki, Emmanouil Foukarakis, Georgia Vlachaki

**Affiliations:** 1 Department of Pediatrics, Venizeleion General Hospital, Heraklion Crete, GRC; 2 Department of Cardiology, Venizeleion General Hospital, Heraklion Crete, GRC

**Keywords:** cardiac manifestations, adolescent, children, pericardial infusion, sars-cov-2

## Abstract

As the COVID-19 pandemic evolves, the medical community continues to report a variety of clinical manifestations of SARS-CoV-2 in the pediatric population. Although younger age groups experience less severe disease, attention is given to the immunologic manifestations of the disease. Pericarditis is a rare cardiac complication of COVID-19 infection. We discuss the first case of delayed presentation of pericarditis following recovery from COVID-19 infection in the pediatric population.

A 15-year-old male adolescent presented to the emergency department (ED) with a two-day history of left-sided, sub-sternal chest pain that worsened during inspiration and a low-grade fever. Twenty days prior to this presentation, the patient experienced fever and was tested positive for SARS-CoV-2. His family history was remarkable for Hashimoto thyroiditis and rheumatoid arthritis, with his mother having experienced 18 episodes of pericarditis during the exacerbations of her disease. RT-PCR for SARS-CoV-2 was negative on this occasion and the serology assay identified positive IgG antibodies against the virus. The ECG was suggestive for pericarditis and the diagnosis was confirmed by the presence of pericardial effusion on ECHO. The rest of the aetiological investigations for pericarditis were negative. In view of the strong family history of autoimmunity, questions were raised in the medical team of our hospital regarding the etiology of his pericarditis and on whether it represented a postinflammatory immune-mediated presentation of SARS-CoV-2 or a new-onset autoimmune disease.

## Introduction

As the COVID-19 pandemic evolves so does our knowledge about the impact of SARS-CoV-2 on the pediatric population. Although COVID-19 generally results in milder disease in children and adolescents, severe illness from COVID-19 can also occur in this age group and might require hospitalization and intensive care unit support [1]. The latter became more obvious after the B.1.617.2 (Delta) variant, which has been the predominant variant of SARS-CoV-2 since late June 2021 globally and has significantly increased the number of Emergency Department visits and hospital admissions in the pediatric population [1]. This higher transmission especially in the vulnerable unvaccinated pediatric population has led to more infections of SARS-CoV-2 in the post-delta era and therefore a broad spectrum of clinical presentations of the virus in the pediatric population [1]. Special attention should be paid to the extrapulmonary manifestations such as cardiac and immunological [2]. Herein, we present a case of postinfectious acute pericarditis in an adolescent fully recovered from a COVID-19 infection. 

## Case presentation

A 15-year-old male adolescent presented to the emergency department (ED) with a two-day history of left-sided, sub-sternal chest pain that worsened during inspiration and a low-grade fever (Tax: 37.8 ^o^C). Twenty days prior to this presentation, the patient experienced fever and was tested positive for SARS-CoV-2 (with an RT-PCR for SARS-CoV-2). He described complete recovery and did not require hospitalization, medications, or supplemental oxygen.

His past medical history was remarkable for four corneal transplantation procedures because of corneal opacity of unknown origin. The family history was remarkable for Hashimoto's thyroiditis and rheumatoid arthritis (RA), with his mother having experienced 18 episodes of pericarditis during the exacerbations of her RA.

On presentation to the ED the patient was afebrile, blood pressure was 114/70 mmHg, heart rate 112 beats/minute and oxygen saturation was 98% in room air. Lung auscultation revealed normal air entry bilaterally, without abnormal sounds. Cardiovascular examination was significant for tachycardia and a systolic murmur of +1/6. No pericardial friction rub, jugular venous distension, or pedal edema was noted. Pulses were 2+ in all four extremities.

Laboratory investigation revealed slightly elevated white blood cells (WBC) of 11,500/μL (neutrophils: 73.9%, lymphocytes: 14.2%, monocytes: 10.9 %) and an elevated erythrocyte sedimentation rate (ESR) of 56 mm (normal values : 0-15 mm) and C-reactive protein (CRP) of 15.3 mg/dL (normal values <0.5 mg/dL). Blood chemistry, including troponin I and ferritin as well as coagulation studies, were all within normal limits (Table 1). His blood culture did not reveal any pathogens. A rapid antigen test and an RT-PCR for SARS-CoV-2 were performed and they were both negative while his serum SARS-CoV-2 IgM and IgG antibodies were both positive.

**Table 1:** Laboratory values WBC: white blood cell count, NEU: neutrophils, LYM: lymphocytes, MONO: monocytes, HCT: Hematocrit, HGB: Hemoglobin, RBC: Red blood cell count, MCV: Mean corpuscular volume, MCH: Mean corpuscular hemoglobin, MCHC: Mean corpuscular hemoglobin concentration, PLT: platelets count, CRP: C-reactive protein, ESR: erythrocyte sedimentation rate, GLU: glucose, Cr: creatinine, Na: sodium, K: potassium, SGOT: serum glutamic-oxaloacetic transaminase, SGPT: serum glutamic pyruvic transaminase, γGT: Gamma-glutamyl transpeptidase, CK: Creatine kinase, LDH: Lactate dehydrogenase, PT: prothrombin time, INR: international normalized ratio, APTT: activated partial thromboplastin clotting time, FIB: fibrinogen, PCT: procalcitonin

Laboratory test	Result	Reference range
WBCs (/mm^2^)	11,500	3,800-10,500
NEU (/μL)	8,500	2,000-7,500
LYM (/μL)	1,600	1,500-3,500
MONO (/μL)	1,300	200-800
HGB (g/dL)	13.60	13.40-17.40
HCT (%)	39.80	41.0-53.8
RBC (M/μL)	4.98	4.78-5.95
MCV (fl)	79.90	82-100
MCH (pg)	27.30	27.70-35.10
MCHC (g/dL)	34.10	29.90-34.90
PLTs (/μL)	249,000	150,000-400,000
ESR (mm/1^st^h)	56	0-15
CRP (mg/dL)	15.32	< 0.5
Ferritin (ng/mL)	244.31	21.81-274.66
PCT (ng/dL)	0.02	0-0.25
GLU (mg/dL)	95	70-110
Urea (mg/dL)	18	15-50
Cr (mg/dL)	0.83	0.7-1.3
Na (mmol/L)	141	136-145
K (mmol/L)	4.80	3.5-5.1
SGOT (U/L)	20	5-35
SGPT (U/L)	20	0-55
Trop-I HS (pg/dL)	2.80	< 34.2
CK (U/L)	109	30-180
LDH (U/L)	194	125-220
PT (sec)	12.90	10.3-13
INR	1.20	0.9-1.2
APTT (sec)	27.90	26-38
FIB (mg/dL)	814.70	200-400
D-Dimmers (mg/L)	1.90	< 0.5
TSH (μUI/mL)	2.48	0.35-4.94
FT4 (ng/dL)	0.88	0.70-1.48
Anti-Tg (IU/mL)	1.03	< 4.11
Anti-TPO (IU/mL)	0.53	< 5.61
C3 (mg/dL)	158	90-180
C4 (mg/dL)	31	10-40
RF (IU/mL)	< 11	< 15
ANA	1:80	
IgA (mg/dL)	245	70-400
IgG (mg/dL)	1,480	700-1,600
IgM (mg/dL)	109	40-230
IgE (IU/mL)	413	0.01-100

**Figure 1:**
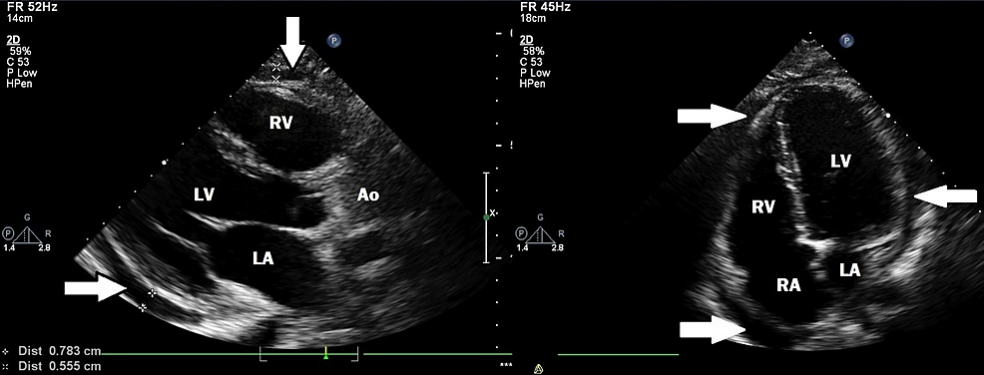
Parasternal long axis and apical four-chamber views of the heart in the left panel and right panel, respectively. Pericardial effusion was noticed with arrows. LV: left ventricle, RV: right ventricle, LA: left atrium, RA: right atrium, Ao: Aorta

Chest radiograph showed an enlarged cardiac silhouette, while ECG showed a depression of the QT segment and an elevation of the ST segment in the leads I, V2-V6. Transthoracic echocardiogram (TTE) revealed a small pericardial effusion all over the heart, sized <1cm in front of the posterior wall of the left ventricle (Figure 1) with otherwise normal cardiac function. With the diagnosis of pericarditis, the patient was admitted and treated with ibuprofen and colchicine was initiated immediately.

Because of the patient’s past medical and family history, further investigations for possible etiologies of pericardial effusion were performed including serologic assays for Coxsackie virus, Epstein-Barr virus, cytomegalovirus, herpes simplex virus 1 and 2, adenovirus, parvo virus, echo virus, mumps, hepatitis B and C, syphilis, HIV and they were all negative. Apart from the mildly positive anti-nuclear antibody (ANA) (1:80), the rest of the autoimmune workup with anti-neutrophilic cytoplasmic antibodies (ANCA), rheumatoid factor (RF), anti-double-stranded DNA (anti-dsDNA), and anti-cyclic citrullinated peptide (anti-CCP), C3 and C4 did not reveal any abnormal findings. His immunoglobins were within a normal range (IgA: 245 mg/dL, IgM: 109 mg/dL, IgG: 1,480 mg/dL, IgE: 413 IU/mL) and his tuberculin test was negative.

**Figure 2:**
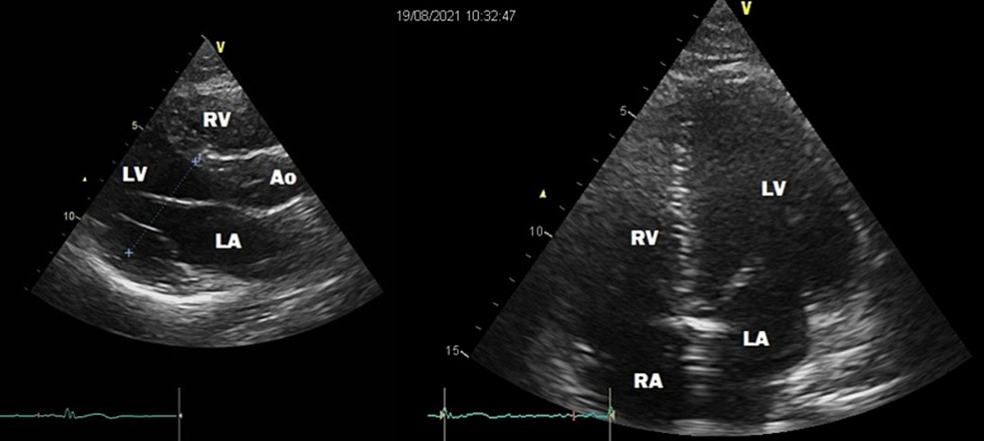
Parasternal long axis and apical four-chamber views of the heart in the left panel and right panel, respectively. Pericardial effusion was disappeared. LV: left ventricle, RV: right ventricle, LA: left atrium, RA: right atrium, Ao: Aorta

During his hospitalization, the patient showed improvement of his symptoms, and repeated TTE did show the reduction of the pericardial effusion within four days (Figure 2). He was discharged on oral ibuprofen and colchicine and followed up regularly in the cardiology clinic with gradual tapering of ibuprofen.

## Discussion

Based on our knowledge, there is an undeniable relationship between SARS-CoV-2 and cardiovascular disease. SARS-CoV-2 is a cardiotropic virus and can affect the heart in many ways. The data about the spectrum of cardiac manifestations οf COVID-19 infection in children are scarce and the most frequently documented cardiac findings in the pediatric population include myocarditis, myocardial dysfunction, pericarditis, and coronary artery involvement in multisystem inflammatory syndrome in children (MIS-C) [3].

Pericardial effusion has been reported in ~5% of adults with COVID-19 occurring at the time of or shortly after diagnosis [4]. There are only four cases of acute pericarditis described in the literature in children and adolescents with an active COVID-19 infection [5-7]. Our case differs in its unusually late and subtle presentation and to the best of our knowledge, it is the first report of a delayed presentation of pericarditis following recovery from COVID-19 infection in the pediatric population. Our patient originally tested positive for SARS-CoV-2 with complete symptomatic recovery and developed a new fever, chest pain, and radiographic evidence of pericardial effusion three weeks later.

Often occurring after viral infections, such as coxsackievirus or herpesvirus, pericarditis may appear weeks after the initial infection [8]. Other risk factors for pericarditis include prior myocardial infarction, trauma, end-stage renal disease, cancer, specifically breast, lung, leukemia, non-Hodgkin’s and Hodgkin’s lymphoma, autoimmune diseases, and medications including hydralazine and procainamide [8]. It is not clear whether pericarditis in our case was just a postinflammatory immune-mediated presentation of SARS-CoV-2 or a new-onset autoimmune disease possibly triggered by the recent viral infection in this susceptible individual. Given the fact that our patient had a mildly positive ANA and that his family history was remarkable for maternal rheumatoid arthritis presenting with recurrent episodes of pericarditis, we could speculate that he could have been vulnerable to virally induced autoimmunity in the forms of autoantibody formation, as well as the development of clinical immune-mediated inflammatory diseases.

Numerous studies have documented the presence of a wide variety of autoantibodies in patients with COVID-19, with antinuclear antibody (ANA) reported in up to 30% of severe cases [9,10]. An overexuberant autoimmune component of COVID-19 has been extensively discussed in the literature but common pathophysiology to explain the variations in clinical presentation has been elusive [11,12]. It is still unclear whether COVID-19 is a viral-induced autoimmune disorder or an imitator of the manifestations of other autoimmune disorders with maybe the same pathophysiology roots [11,12].

## Conclusions

It is important to remain vigilant for extrapulmonary manifestations of the disease in pediatric patients with delayed nonspecific symptoms after COVID-19 infection. Regardless if it represents a postinflammatory immune-mediated presentation of SARS-CoV-2 or a new-onset autoimmune disease, pericarditis should be included in the differential diagnosis of patients presenting with unspecific symptoms in the period following recovery from COVID-19 infection.
